# Habitual Behavior Is Mediated by a Shift in Response-Outcome Encoding by Infralimbic Cortex

**DOI:** 10.1523/ENEURO.0337-17.2017

**Published:** 2018-01-03

**Authors:** Jacqueline M. Barker, W. Bailey Glen, David N. Linsenbardt, Christopher C. Lapish, L. Judson Chandler

**Affiliations:** 1 Department of Neuroscience, Medical University of South Carolina, Charleston, SC 29425; 2 Department of Psychology, Indiana University Purdue University Indianapolis, Indianapolis, IN 46202-3275

**Keywords:** goal-directed behavior, habit, infralimbic, prefrontal cortex

## Abstract

The ability to flexibly switch between goal-directed actions and habits is critical for adaptive behavior. The infralimbic prefrontal cortex (IfL-C) has been consistently identified as a crucial structure for the regulation of response strategies. To investigate the role of the IfL-C, the present study employed two validated reinforcement schedules that either promote habits or goal-directed actions in mice. The results reveal that information about action-outcome relationships is differentially encoded in the IfL-C during actions and habits as evidenced by encoding of behavioral outcomes during goal-directed actions that is lost during habits. Optogenetic inhibition of the IfL-C selectively at press during habitual behavior (when firing rates are reduced during unreinforced goal-directed actions) resulted in restoration of sensitivity to change of action-outcome contingency. These results reveal a novel functional mechanism by which IfL-C promotes habitual behavior, and provide insight into strategies for the treatment and prevention of pathological, inflexible behavior common in neuropsychiatric illness.

## Significance Statement

The loss of the ability to flexibly regulate reward seeking is associated with multiple neuropsychiatric illnesses, including addiction. Here, we demonstrated that during flexible, goal-directed actions, the infralimbic prefrontal cortex (IfL-C) encodes reinforcer delivery information. In contrast, during the performance of inflexible, habitual behavior, IfL-C encoding of action-outcome contingency information was attenuated. Inhibition of IfL-C selectively during this task epoch restored sensitivity to changes in contingency, suggesting that IfL-C activity can determine response strategy. These data provide novel insight into the precise neural functions that underlie the development and maintenance of habitual behavior and are expected to inform the development of treatment strategies for deficits in cognitive control over behavior.

## Introduction

Maladaptive behaviors, including overeating, drug abuse, and perseverative obsessive-compulsive rituals, are characterized by the inability to update actions when goals change (Balleine and O’Doherty, 2010; Sjoerds et al., 2013; Barker and Taylor, 2014; Gillan et al., 2014, 2015; Everitt and Robbins, 2015). These inflexible behaviors likely consist of habits in which actions are no longer performed in relation to their outcome. Extensive research has focused on elucidating the circuitry underlying habit formation and expression. While it is generally thought that subregions of the prefrontal cortex mediate cognitive control of actions, the infralimbic prefrontal cortex (IfL-C) appears to sub-serve habitual, stimulus-driven reward seeking (Killcross and Coutureau 2003; Coutureau and Killcross 2003; Smith et al., 2012; Smith and Graybiel 2013; Barker et al., 2014). The IfL-C is critical for both the acquisition (Killcross and Coutureau, 2003) and expression (Coutureau and Killcross, 2003; Smith et al., 2012) of habitual behavior. Lesioning the IfL-C prevents the development of habitual reward seeking even with extensive training (Killcross and Coutureau, 2003), while inactivation of IfL-C after the development of habitual behavior can restore goal-directed actions (Coutureau and Killcross, 2003; Smith et al., 2012). Interestingly, IfL-C is also critical for the extinction of drug seeking and fear behaviors (Peters et al., 2009). Collectively, these data suggest a critical role of the IfL-C in the regulation of contingency-mediated behaviors by promoting both habitual behavior and extinction, potentially by suppressing previously established contingencies (Barker et al., 2014).

While lesion and pharmacological studies consistently implicate an important role for IfL-C in response strategy, it is not known how neurons within the IfL-C encode actions and outcomes and whether these representations are different when behaviors are habitual or goal directed. Therefore, the present study took advantage of two different instrumental training schedules known to produce different response strategies (Adams and Dickinson, 1981; Gremel and Costa, 2013), a habit-promoting schedule and an action-promoting schedule, to determine how the IfL-C encodes actions and outcomes during the performance of habitual versus goal-directed behaviors. Through the use of this behavioral model, task-dependent modulations of neural activity during key epochs of behavior were assessed that included the interval when information on action-outcome contingencies was provided (i.e., during reinforcer delivery after a lever press) and during intervals where reward value information was available (i.e., during consumption of the reinforcer). By performing these analyses within subjects, alterations in neural activity patterns can be identified during encoding of contingency and outcome information that are mediated by response strategy and by training and schedule. These findings provide a greater understanding of the neural activity patterns that emerge in the IfL-C during the expression of habitual reward seeking, which is critical for identifying the mechanisms underlying the transition from goal-directed actions to habits. These data provide novel insight for the development of treatments and prevention strategies for dysregulated behavior common in neuropsychiatric illnesses.

## Materials and Methods

### Subjects

Adult male C57BL/6J mice (>10 weeks of age) from The Jackson Laboratory were used in these studies in accordance with the University Institutional Animal Care and Use Committee guidelines. Mice were housed in a vivarium with a reverse 12/12 h light/dark cycle, and all experimental testing was performed during the dark cycle. For behavioral testing, mice were food restricted to 90% of their free-feeding weights. Water was available ad libitum for the duration of the study.

### Multielectrode array implantation

Two types of probes were used. For 6 mice, custom made probes consisting of 14 microwires for recording spike activity were fabricated as previously described (Lapish et al., 2008). The spatial configuration was determined by feeding polyimide coated tungsten wires (25 µm) through a guide array made from Protomid polyimide tubes (OD: 120 µm) assembled side by side in a 7 × 2 arrangement. The day of the unilateral surgical implantation of the array into the IfL-C (7 × 2, AP +1.3 to AP +2.0 ML ±0.2–0.4, DV −3.0), wires were cut to 3.0 mm in length from the end of the guide tubes. Two additional mice were implanted with probes purchased from Innovative Neurophysiology with wires targeting IfL-C in an 8 × 2 arrangement (35-μm diameter, 150-μm spacing). Surgeries were performed under isoflurane anesthesia with Rimadyl administered both pre- and postoperatively. After implantation, the probes were secured to the skull using Metabond and dental cement. To confirm wire placement at the end of the experiments, mice were deeply anesthetized with isoflurane and microlesions were created at electrode tips by current injection (100 μA, 1 s per wire) before transcardial perfusion with 4% paraformaldehyde (Fig. 2).

### Instrumental conditioning chambers

A standard instrumental chamber housed within a sound-attenuating box was used for these experiments (Med-Associates). The side walls of the chamber were made of stainless steel panels and the front door, ceiling and back wall were made of clear Plexiglas. The ceiling had an opening to allow access of MEA cable that tethers the headstage to the implanted MEA probe. The chamber had a house light located at the top of the middle panel on the right wall, above a modified magazine equipped with a photo-beam sensor. Retractable levers were extended during relevant sessions on either side of the magazine and liquid reinforcers were delivered by syringe pump to the magazine. A fan provided ventilation and background white noise.

### Instrumental training

**Figure 1.**
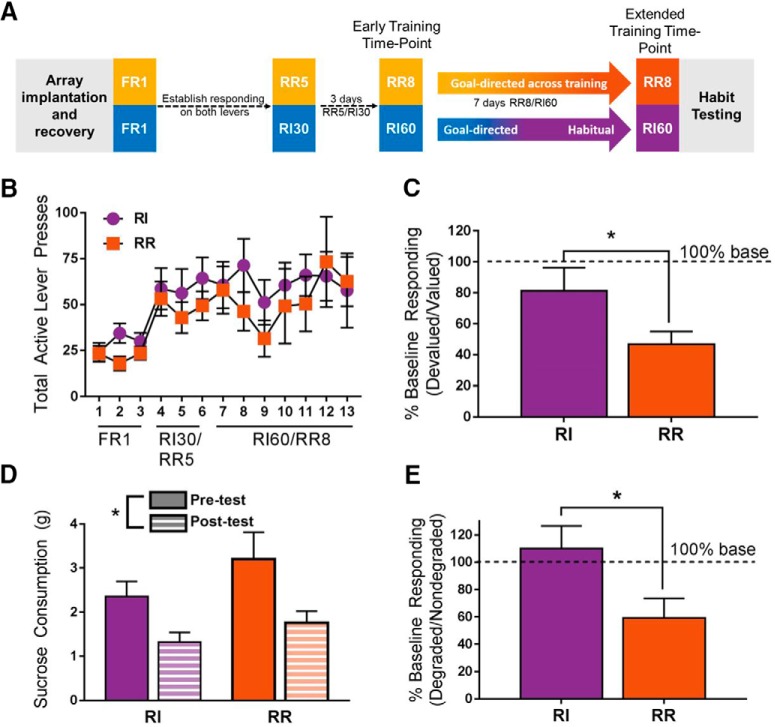
[AQ5] Experimental design and assessment of response strategy. ***A***, Mice were implanted with multielectrode array probes and, following recovery from surgery, were food restricted and trained to self-administer sucrose on two levers on an FR1 schedule. After establishing responding on each lever, schedules were transitioned to RR5 on one lever and RI30 on the other lever. The order of lever presentation was counter-balanced. After 3 d on the RR5/RI30 schedule, mice were transitioned to the RR8/RI60 schedule. Recordings for the early training time period took place at the first and second sessions of RR8/RI60 and recording for the extended training time period were the final two RR8/RI60 sessions before habit testing. ***B***, While no differences in response rate were observed between an RR and RI schedule, responding significantly increased across day of training (*p* < 0.01). Importantly, no differences in responding were observed between the RR and RI levers (*p* = 0.288). ***C***, Normalized responding during the RR test session was significantly lower than responding during the RI test session during specific satiety outcome devaluation. ***D***, Outcome devaluation reduced sucrose consumption such that mice consumed less sucrose in the 1 h “post-test” session than in the “pre-test” session. ***E***, During the contingency degradation test session, responding was significantly lower during the RR session than during the RI session. Together, these data demonstrate that responding on the RR lever remained goal-directed at a time point at which responding on the RI lever was habitual; *p < 0.05. Data represent mean ± SEM.

After recovery from surgery, mice were food restricted and trained to self-administer 10% sucrose via two levers. Each day, mice had a 30-min training session, and each individual lever was presented separately and was only available for 15 min. The order of lever availability was alternated daily. A press on either lever was initially reinforced on a fixed ratio 1 (FR1) schedule in which each lever press resulted in a single delivery of 20 µl of sucrose. Mice were not tethered to the recording system until acquisition of the FR1 response, but were subsequently tethered for all training and testing sessions. After acquisition, the reinforcement schedule on the lever was transitioned to either a random ratio (RR) 5 or a random interval (RI) 30 schedule (Gremel and Costa, 2013). On the RR5 schedule, the number of lever presses made controlled reinforcer delivery. On average, the fifth response was reinforced, but the program software randomly generated the actual response requirement. On the RI 30 schedule, the first lever pressed after a randomly determined interval (averaging 30 s) has elapsed was reinforced. The number of lever presses made on the RI schedule was not related to reinforcer delivery. After the 3^rd^ session, schedules were then transitioned to RR8 and RI60. The response schedule assigned to each lever was consistent across training for each animal (i.e., left lever was always the “interval” lever and right lever was always the “ratio” lever; Fig. 1*A*). These response schedules were selected so that response rate and reinforcement rate were matched across RR and RI responding (Fig. 1*B*).

### Habit testing

#### Outcome devaluation

The expression of habitual behavior was assessed using a specific satiety outcome devaluation procedure. One hour prior to a testing session, mice were placed in a novel environment and allowed to consume the 10% sucrose reinforcer. After 1 h, mice were tested in the same instrumental conditioning chamber where training took place. All behavioral self-administration sessions and habit testing took place in the same operant chamber. These extinction test sessions were only 10 min in duration in order to limit extinction learning. Lever presses were recorded and the pump was turned on at the same rate as during the last training session, but no sucrose was available. After testing, mice again received 1-h access to 10% sucrose to confirm devaluation. All mice were tested for sensitivity to outcome devaluation on both levers, and the order of testing was counter-balanced. Between test sessions, mice were retrained in a standard RR8/RI60 session. Responding was normalized to each individual’s performance on a session in which they were not sated and the contingency was intact. Importantly, this does not control for satiety and it is possible that effects observed in the extinction session may be related to satiety independent of reinforcer devaluation. In addition, it is possible that responding on RR versus RI schedules was differentially sensitive to extinction, which may further contribute to differences observed during this test session.

#### Contingency degradation

Habitual behavior was further assessed using a contingency degradation measure in which conditions were identical to training conditions, except that the 10% sucrose reinforcer was delivered on a noncontingent schedule. Here, reinforcer delivery was determined by the number of reinforcers each animal earned during a session in which the RR or RI contingency was intact. Delivery was spaced equivalently across the 15-min session. Responses on the extended lever were recorded but did not have an outcome. Testing for the RR or RI lever occurred on separate days, separated by a “retraining” session in which the contingencies were intact. Responding during a contingency degradation test session was compared to a test session in which the animal was tethered but the contingency was intact.

### Neurophysiological data collection and analysis

Following recovery and acquisition of the FR1 schedule, mice were connected to a custom recording system that consisted of Plexon and Multichannel Systems (MCS) hardware. The animals were connected via a Plexon headstage (HST/16o25) and a 16-channel cable custom fabricated by Omnetics. This cable was attached to an MCS signal collection box, and the data were acquired by MCRack at 40 kHz. A TTL pulse driven by the Med Associates hardware enabled synchronization between behavioral measures collected by Med Associates (RRID:SCR_014721) and electrophysiological measures collected in MCRack. The pulse was delivered at the start of the behavioral procedure, as well as at each lever press, and was detected in MCS hardware. Recording files for both the behavior and the MEA recordings were imported and merged in Matlab (RRID: SCR_001622). Data from five animals were analyzed from two sessions, an early training time point, at which behavior was expected to be goal directed on both schedules, and an extended training time point at which mice responded habitually on the RI schedule but not on the RR schedule.

### Spike sorting

Sorting was performed using Plexon Offline Sorter. Preprocessing steps for spike detection and spike sorting included automated identification and zeroing of periods with an occasional amplifier overload artifact, common average referencing of functional channels (Ludwig et al., 2009), and zero-phase band pass filtering [0.3–5 kHz Bessell with Matlab function filtfilt()]. A negative threshold of 3 times the root mean squared voltage for each channel and aligned to the maximum local negative peak was used to identify potential spikes. Clearly isolated units were identified via examination of various features and component views of the waveforms. This yielded 136 distinct units from the early training sessions and 115 units from the extended training sessions (Table 1). Total units analyzed under different conditions (i.e., isolated lever presses versus consummatory behavior) differed in part due to the ability to isolate behavioral events during the self-paced task. After sorting, Matlab was used to exclude any possible duplicate units (e.g. identical spike times). Spikes were subsequently binned at 100 ms and smoothed across five bins using a moving window average function (Matlab function smooth.m). The smoothed composite bins surrounding events of interest were then identified.

**Table 1.** Total datasets and cells analyzed from individual animals

Mouse	Datasets: early training	Datasets: extended training	Cells early	Cells extended
A	2	1	22	7
B	2	1	13	6
C	2	1	21	11
D	2	2	21	20
E	2	2	13	16
F	2	2	13	15
G	2	2	17	22
H	2	2	16	18
Total:			136	115

A maximum of 136 units were included in analyses at the early time point, and a maximum of 115 at the extended training time point. Actual cells included in analysis at distinct task epochs varied based on behavior and data contamination as a result of performance.

### Optical inhibition of IfL-C

To determine the role of IfL-C firing at lever press in response strategy selection, mice received an injection of an AAV expressing an inhibitory opsin (AAV2-hSyn-eArch3.0-EYFP; University of North Carolina Vector core; RRID:SCR_002448) into the IfL-C (AP +1.7, ML ±1.25, DV −3.12 at 15° angle, targeting AP +1.7, ML ±0.4, DV −3.0; 0.2 μl/side). In the same surgery, optic fibers were implanted targeting the same site. Two weeks after surgery, mice were food restricted to 90% of their free feeding weights and trained to self-administer 10% sucrose using the RR/RI procedure described above. After the final day of RR8/RI60 training, mice were assigned to one of three groups for contingency degradation testing, either no inhibition, inhibition at press, or unpaired inhibition. For the no inhibition group, mice were tethered during both a degraded and nondegraded test session, but no light was delivered. For both conditions in which light was delivered, mice received continuous light for 0.5 s at 5 mW at 532 nm. In the “inhibition at press” condition, light was delivered at all presses (reinforced or unreinforced) for 0.5 s. In the “unpaired” condition, light was delivered for 0.5 s at intervals that were explicitly unpaired with either lever presses or reinforcer delivery. While we did not record neural activity during optical inhibition, we expect that the use of this protocol in which light is delivered for 0.5 s will disrupt neural activity during the 0.5 s following responding (Chow et al., 2010). However, we cannot rule out the possibility that a “rebound”-like effect resulted in firing following this period of inhibition that may contribute to the ability to use contingencies to guide behavior. The order of degraded and nondegraded test sessions was counterbalanced. To control for effect of IfL-C inhibition on baseline responding, mice were assigned to the same condition in a nondegraded control session in which the RI schedule was intact to compare to the degraded test session in which the action-outcome association was no longer in place.

To demonstrate that IfL-C inhibition at press did not result in a reduction in responding during the contingency degradation test session by facilitating extinction, mice were retrained for 5 d on the RR8/RI60 protocol and tested in an extinction session in which the outcome was not delivered. Laser was delivered under the same conditions described above.

### Statistical analyses

**Figure 2.**
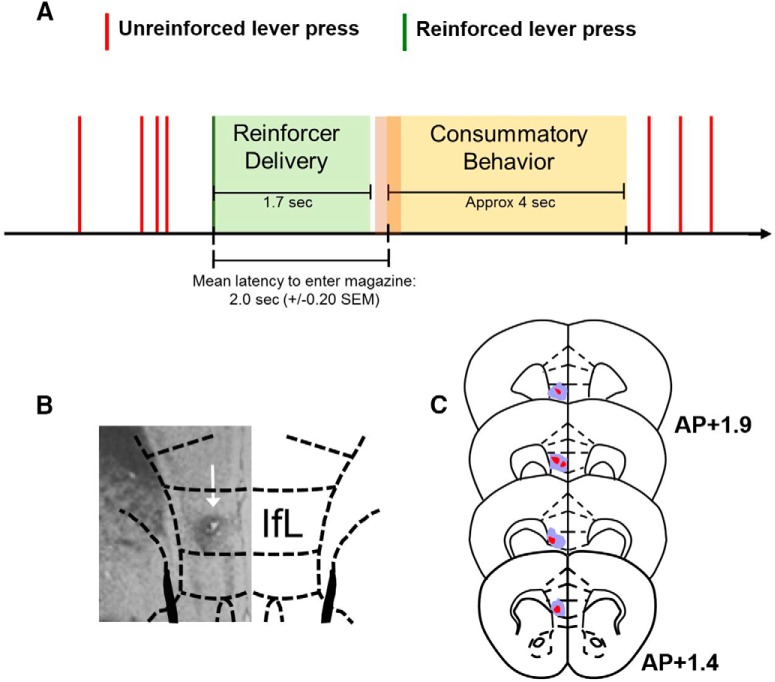
Experimental and recording session time line. ***A***, Illustration of recording session time line. Unreinforced presses (indicated by vertical red lines) were those that did not result in reinforcer delivery. Only isolated unreinforced presses were included in analyses. After reinforced presses (indicated by vertical green line), reinforcer delivery began immediately and lasted 1.7 s. The mean time of consummatory behavior onset was 2.0 s (±0.203 s, SEM) following a reinforced lever press. While the mean time of magazine entry occurred after the termination of the reinforcer delivery epoch, the initiation of consummatory behavior was self-paced and overlap between the reinforcer delivery epochs and consummatory behavior epochs was variable. For analysis of reinforced presses, the pre-press interval consisted of 0.5 s before the initiation of press. The post-press epoch was defined as 0.5 s following the lever press. The reinforcer delivery epoch was defined as the remainder of the “pump on” interval. ***B***, Mice were implanted with multielectrode array probes and, following recovery from surgery, were trained to self-administer sucrose in the RR/RI protocol (Fig. 1). Shown is an example of electrolytic lesions of the recording site. ***C***, Placement of multielectrode arrays within IfL-C for all mice in the study. Blue shaded region represents the maximal size of the lesioned area within each of the anatomic sections shown, while red represents the smallest lesion area within each section.

To investigate firing rate changes across reinforced lever presses, 100-ms bins were taken from the −3- to +10-s time period surrounding the “pump-on” epoch, which occurred at the time of a reinforced lever press (Fig. 2). To determine the effects of reinforcer consumption on firing rate in IfL-C, the onset of consumption was estimated to be the time of the first magazine entry (measured by beam break) occurring within 4 s of a reinforced press. Bins were taken from −3- to +15-s time period following the beam break. While this resulted in a variable degree of overlap between the period analyzed during reinforcer delivery and the epoch defined as reinforcer consumption, overlap between magazine entry and reinforcer delivery was not mediated by either training time point or reinforcer schedule.

To determine how lever presses in the absence of reinforcer delivery impacted firing rate surrounding unreinforced presses, bins were taken from the −3- to +3-s time period for unreinforced presses, which were absent any other presses or beam breaks within the 6s period, to obtain isolated unreinforced presses. Event time bins that were contaminated by overload artifact were removed from analysis. The mean firing rate for each unit was calculated and the *z* score taken across the entire bin. These exclusion criteria were applied only to isolated, unreinforced lever presses. Overall means represent the mean of firing rate z-scores for each combination of schedule and training time point. D-prime (d′) analysis, which represents the absolute value of the mean differences in firing rate between epochs divided by the pooled standard deviation, was used to identify cells significantly modulated across task epochs. For reinforcer delivery and unreinforced press intervals, the −3- to 0-s time period before the event was compared to the 0- to +3-s period after the event. For reinforcer consumption, the 0- to +5-s time period after the event was compared to the +8s to +13s period after the event. D′ was calculated for each unit, and significance thresholds were determined for each neuron using surrogate datasets. The probability density function (PDF) for each neuron was randomly shuffled 500 times, and the mean d′ of each of the 500 permutations were used to calculate 95% confidence intervals. Cells were then grouped as those that exhibited significant increase, decrease, or no change in firing. Units were sorted by the direction and magnitude of selectivity and plotted as a heat map, and the means of increasing and decreasing units were plotted as line graphs.

For all datasets, statistical analysis was performed in SPSS (RRID:SCR_002865). For population distribution and proportional tests, analysis was performed using χ^2^ test of independence and assessment of proportional distribution. Firing rate data were analyzed using repeated measures ANOVA (rmANOVA) as appropriate, and *post hoc* analyses were performed using Sidak’s correction for multiple correction. rmANOVA were corrected using Greenhouse-Geisser in instances where unequal variance was observed (Table 2).

**Table 2.** Statistical table

	Data structure	Type of test	Power
a	Normal	rmANOVA	Main effect of day: 0.967 Main effect of schedule: 0.170 Interaction: 0.211
b	Normal	rmANOVA	Main effect of day: 1.0 Main effect of schedule: 0.358 Interaction: 0.492
c	Non-normal	Wilcoxon	*p* < 0.05
d	Normal	rmANOVA	Main effect of devaluation: 0.738 Main effect of schedule: 0.281 Interaction: 0.117
e	Normal	Paired *t* test	
f	Unequal variance (epoch violates Mauchly’s)	rmANOVA with Greenhouse-Geisser	Main effect of schedule: 0.54 Main effect of epoch: 0.981 Interaction: 0.131
g	Unequal variance (epoch violates Mauchly’s)	rmANOVA with Greenhouse-Geisser	Main effect of schedule: 0.157 Main effect of epoch: 0.374 Interaction: 0.591
h	Categorical data	χ^2^	0.967
i	Categorical data	χ^2^	0.992
j	Normal	rmANOVA	Main effect of schedule: 0.112 Main effect of epoch: 1.0 Interaction: 0.913
k	Unequal variance (epoch violates Mauchly’s)	rmANOVA with Greenhouse-Geisser	Main effect of schedule: 0.107 Main effect of epoch: 0.991 Interaction: 0.526
l	Normal	rmANOVA	Main effect of schedule: 0.138 Main effect of epoch: 1.0 Interaction: 0.065
m	Normal	rmANOVA	Main effect of schedule: 0.379 Main effect of epoch: 1.0 Interaction: 0.706
n	Unequal variance (epoch violates Mauchly’s)	rmANOVA with Greenhouse-Geisser	Main effect of schedule: 0.617 Main effect of epoch: 0.156 Interaction: 0.519
o	Normal	rmANOVA	Main effect of schedule: 0.050 Main effect of epoch: 0.888 Interaction: 0.998
p	Normal	rmANOVA	Main effect of schedule: 0.384 Main effect of epoch: 0.466 Interaction: 0.211
q	Normal	rmANOVA	Main effect of schedule: 0.262 Main effect of epoch: 0.925 Interaction: 0.104
r	Normal	ANOVA	Main effect of schedule: 0.088 Main effect of time point: 0.176 Interaction: 0.051
s	Non-normal	Wilcoxon	
t	Non-normal	Wilcoxon	
u	Non-normal	Wilcoxon	
v	Non-normal	Wilcoxon	
w	Normal	ANOVA	Main effect of schedule: 0.232 Main effect of time point: 0.050 Interaction: 0.061
x	Normal	rmANOVA	Main effect of schedule: 0.300 Main effect of epoch: 0.150 Interaction: 0.652
y	Categorical data	Z score two-population proportions	0.800
z	Categorical data	Z score two-population proportions	0.057
aa	Normal	rmANOVA	Main effect of schedule: 0.052 Main effect of epoch: 0.399 Interaction: 0.896
ab	Categorical data	Z score two-population proportions	0.78
ac	Categorical data	Z score two-population proportions	0.56

ad	Normal	rmANOVA	Degradation: 0.919 Light: 0.056 Interaction: 0.821
ae	Normal	rmANOVA	Session: 0.976 Light: 0.067 Interaction: 0.054
af	Unequal variance (epoch violates Mauchly’s)	rmANOVA with Greenhouse-Geisser	Main effect of schedule: 0.405 Main effect of epoch: 0.961 Interaction: 0.120
ag	Unequal variance (epoch violates Mauchly’s)	rmANOVA with Greenhouse-Geisser	Main effect of schedule: 0.277 Main effect of epoch: 1.0 Interaction: 0.409
ah	Unequal variance (epoch violates Mauchly’s)	rmANOVA with Greenhouse-Geisser	Main effect of schedule: 0.103 Main effect of epoch: 0.963 Interaction: 0.740
ai	Unequal variance (epoch violates Mauchly’s)	rmANOVA with Greenhouse-Geisser	Main effect of schedule: 0.567 Main effect of epoch: 0.541 Interaction: 0.239
aj	Unequal variance (epoch violates Mauchly’s)	rmANOVA with Greenhouse-Geisser	Main effect of schedule: 0.746 Main effect of epoch: 1.0 Interaction: 0.103
ak	Normal	rmANOVA	Main effect of schedule: 0.052 Main effect of epoch: 0.364 Interaction: 0.084
al	Unequal variance (epoch violates Mauchly’s)	rmANOVA with Greenhouse-Geisser	Main effect of schedule: 0.129 Main effect of epoch: 1.0 Interaction: 0.319
am	Unequal variance (epoch violates Mauchly’s)	rmANOVA with Greenhouse-Geisser	Main effect of schedule: 0.206 Main effect of epoch: 0.237 Interaction: 0.125

## Results

### Mice perform actions and habits based on reinforcement schedule

To investigate how neural activity in the IfL-C is altered during the expression of habitual behavior, a behavioral protocol was used that enabled within-session assessment of neural activity during goal-directed actions and habits (Adams and Dickinson, 1981; Gremel and Costa, 2013; Figs. 1*A*, 2*A*). To this end, adult male C57BL/6J (B6) mice were implanted with multielectrode arrays (MEA) targeting the IfL-C (*n* = 10; two mice were excluded from recording analyses due to inaccurate electrode placements; Fig. 2*B*,*C*) and trained to self-administer 10% sucrose solution via two levers, each with a different schedule of reinforcement known to differentially impact the expression of habitual behavior: an action-promoting (RR) and a habit-promoting (RI) schedule (Fig. 1*A*). Responding on RR schedules has been shown to remain goal directed (sensitive to outcome devaluation and changes in action-outcome contingency) over extended training, while responding on an RI schedule rapidly transitions to outcome-insensitive habits (Adams, 1982; Gremel and Costa, 2013). Schedules were selected to match response rate and reinforcement rate across RR and RI responding. Analyses confirmed there were no differences in response rate (Fig. 1*B*_a_) or in reinforcer delivery between the RR and RI schedules. rmANOVA (day of training × schedule) revealed a significant main effect of day of training on lever pressing [*F*_(12,84)_ = 2.664, *p* < 0.01]. *Post hoc* analyses confirmed that lever pressing increased across days of training. No differences in responding were observed between the RR and RI levers [no main effect of lever, *F*_(1,7)_ = 1.322, *p* = 0.288_b_]. No interaction between day of training and schedule was observed [*F*_(12,84)_ = 0.402 *p* = 0.959]. rmANOVA (day of training × schedule) indicated a main effect of day on reinforcers earned [*F*_(12,72)_ = 26.68, *p* < 0.001]. No main effects of lever [*F*_(1,7)_ = 3.583, *p* = 0.107] or day × lever [*F*_(12,72)_ = 0.932, *p* = 0.521] were observed.

Habitual behaviors, by definition, are insensitive to changes in outcome value and action-outcome contingencies (Everitt and Robbins, 2015). After extended training, response strategies were assessed using a specific satiety outcome devaluation procedure in which mice received ad libitum access to sucrose solution for 1 h before testing in extinction. Analysis of responding after devaluation indicated that responding on the RR schedule was lower than responding on the RI schedule following specific satiety devaluation (Wilcoxon signed-rank test, z = 2.197, *p* < 0.05_c_), consistent with a habitual response strategy on the RI lever while responding remained goal directed on the RR schedule (Fig. 1*C*). To confirm outcome devaluation was achieved as a result of the ad libitum consumption session before the extinction test, the amount of sucrose consumed was assessed before and after the 10-min habit test. A significant reduction in sucrose consumption was observed for both the RI and RR schedules [rmANOVA indicated a main effect of devaluation *F*_(1,4)_ = 12.027, *p* < 0.05; Fig. 1*D*_d_], but no effect of schedule was observed (*p* = 0.391). In calculating these volumes, two instances in which weights could not be obtained across all conditions due to leakage or spillage were excluded from analyses. Importantly, a separate test session in which animals were sated on an alternative reward were not performed, and so it is necessary to consider that general effects of satiety may differentially impact responding on RR versus RI schedules.

Response strategy selection was also confirmed using a contingency degradation test paradigm in which the action-outcome contingency was degraded by providing noncontingent reinforcement at a rate determined by each individual animal’s performance (Barker et al., 2013; Barker et al., 2014). Again, responding was reduced on the RR schedule and was significantly lower than responding on the RI schedule during test [paired *t* test, *t*_(8)_ = 3.42, *p* < 0.05; Fig. 1*E*; one mouse was excluded from the contingency degradation test session because of technical issues during test_e_] consistent with habitual responding on the RI schedule, while responding on the RR schedule remained goal directed.

### IfL-C encoding of outcome is attenuated during habitual behavior

**Figure 3.**
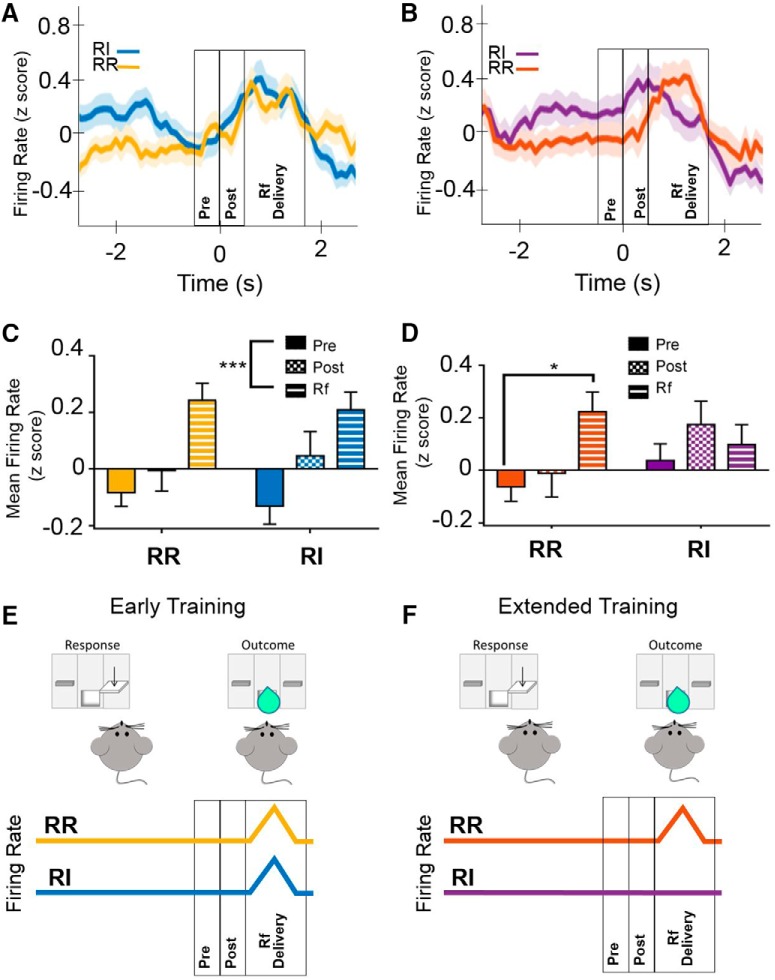
IfL-C firing rate was mediated by response strategy. ***A***, Firing rate surrounding a reinforced lever press at the early training time point (i.e., goal directed on both the RR and RI levers). Time 0 represents the time of lever press. Reinforcer delivery lasted for 1.7 s following lever press. ***B***, Firing rate surrounding a reinforced lever press at the extended training time point. At this time point, responding is habitual on the RI lever but remains goal directed on the RR lever. Data in ***A***, ***B*** represent the mean ± SEM, indicated by shaded area for each line. ***C***, In early training, the mean firing rate during the reinforcer delivery epoch was higher than firing rate during the pre-press interval. This effect was not mediated by reinforcement schedule. ***D***, During responding on the RR schedule (i.e., in a goal-directed manner), firing rate was significantly higher during the reinforcer delivery epoch than during the pre-press interval. This relationship was lost during habitual responding. ***E***, At an early training time point, IfL-C firing rate is modulated by outcome delivery in both action-promoting (RR) and habit-promoting schedules (RI). ***F***, After extended training, IfL-C firing rate is only modulated by outcome delivery in the action-promoting (RR) schedule, and this effect is attenuated in the habit-promoting condition (RI).

To investigate the effects of reinforcement schedule and training on task-dependent alterations in neuronal activity in the IfL-C, data from sessions when animals were presumed to be responding in a goal-directed manner on both the RR and RI schedules (i.e., the first and second RR8/RI60 session) were compared to sessions when behavior was habitual on the RI lever but remained goal directed on the RR lever (i.e., final two RR8/RI60 sessions; Fig. 1). To investigate the neural processes that mediate goal-directed behaviors, analyses of electrophysiological data focused on several key epochs, the “pre-press” interval consisting of the 0.5 s before lever press, the “post-press” interval consisting of the 0.5 s after the lever press, and the “reinforcer delivery” interval consisting of the 1.7-s duration during which the pump delivered sucrose to the well (Table 1). At the early training time point, comparison of the post-press and reinforcer delivery epochs indicated a main effect of time only [unequal distribution of variance; *F*_(1.724,177.6)_ = 9.382, *p* < 0.01; Fig. 3*A*,*C*_f_]; main effect of schedule and epoch × schedule interaction were nonsignificant [*F*_(1,177.6)_ = 0.034, *p* = 0.853 and *F*_(1.89,177.6)_ = 0.527, *p* = 0.581, respectively). *Post hoc* analyses indicated that firing rate at the pre-press interval was significantly lower than firing rate during the reinforcer delivery epoch (*p* < 0.01), while the rate during the post-press interval did not significantly differ from the firing rates during either the pre-press (*p* = 0.134) or reinforcer delivery epoch (*p* = 0.074). In contrast, at the extended training time point at which responding was habitual on the RI schedule, analyses of epochs indicated an epoch × schedule interaction [unequal distribution of variance; *F*_(1.985,168.72)_ = 3.098, *p* < 0.05], but no significant main effects of schedule or epoch [*F*_(1,170)_ = 0.917, *p* = 0.341; *F*_(1.775,168.72)_ = 1.946, *p* = 0.151, respectively; Fig. 3*B*,*D*_g_). *Post hoc* analyses indicated that after extended training on the RR (goal-directed) schedule, firing rate during the reinforcer delivery epoch was significantly higher than pre-press firing (*p* < 0.05), consistent with the pattern observed during both the RR and RI levers during early training. In contrast, this pattern was absent during responding on the RI (habitual) lever. These data suggest that encoding of outcome delivery is present in IfL-C during goal-directed actions, but is attenuated during habitual behavior (Fig. 3*E*,*F*).

**Figure 4.**
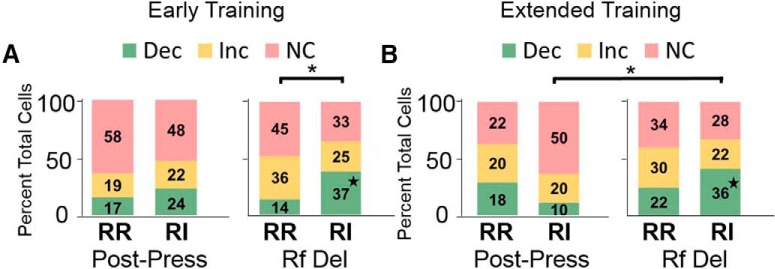
Task-epoch specific modulation of cells. ***A***, At the early training time point, no significant differences in the proportion of cells modulated by lever press were present (left panel). In contrast, during the reinforcer delivery epoch, a greater proportion of cells showed a reduction in firing rate during the RI condition than during the RR condition (*p* < 0.05). ***B***, At the extended training time point, no significant differences in the number or proportion of cells responding were present between RR and RI responding at the post-press (left panel) or reinforcer delivery (right panel) epochs. In contrast, for the RI schedule, the distribution of significantly modulated cells was distinct between the post-press and reinforcer delivery intervals. In the RI condition, a greater proportion of cells showed a decrease in firing rate during the reinforcer delivery epoch (*p* < 0.05). Dec, decreased firing rate, Inc, increased firing rate; NC, no change in firing rate; * indicates significant χ^2^ result; ★ indicates significant differences in *z* score comparison of population proportions.

Cells that were significantly modulated during the press and reinforcer delivery epochs were identified using d*'* analysis. The distribution of cells that were significantly modulated did not differ significantly based on reinforcement schedule during the post-press and reinforcer delivery epochs during goal-directed behavior. Specifically, at the early training time point, a comparable number of cells were modulated during the post-press interval (χ^2^ test of independence; χ^2^ = 3.921, *p* = 0.848; Fig. 4*A*, left panel). In contrast, reinforcement schedule mediated cell modulation during the reinforcer delivery period early in training (χ^2^ test of independence; χ^2^ = 14.20, *p* < 0.01; Fig. 4*A*, right panel_h_). Comparison of proportion of cells modulated (two-tailed *z* score population proportion) indicated a greater proportion of cells exhibited a reduction in firing rate during the reinforcer delivery when mice were responding on an RI (habit-promoting) schedule than on an RR (action-promoting) schedule, likely before the development of habitual behavior.

After extended training, when responding is expected to be habitual on the RI lever, the distribution of significantly modulated cells was different between the press and reinforcer delivery intervals (χ^2^ = 10.54, *p* < 0.05; Fig. 4*B*_i_). Comparison of the proportion of cells modulated (two-tailed *z* score population proportion) indicated an increase in cells showing a reduction in firing rate during the reinforcer delivery epoch as compared to the post press epoch (z = 4.223, *p* < 0.05, green block; Fig. 4*B*), but no difference in the proportion of cells showing an increase in firing rate during these task epochs (z = 0.0861, *p* = 0.928; yellow block; Fig. 4*B*). These data indicate that, when behavior is habitual, a decrease in neural firing is observed during reinforcer delivery that may reflect a loss of goal encoding in IfL-C.

**Figure 5.**
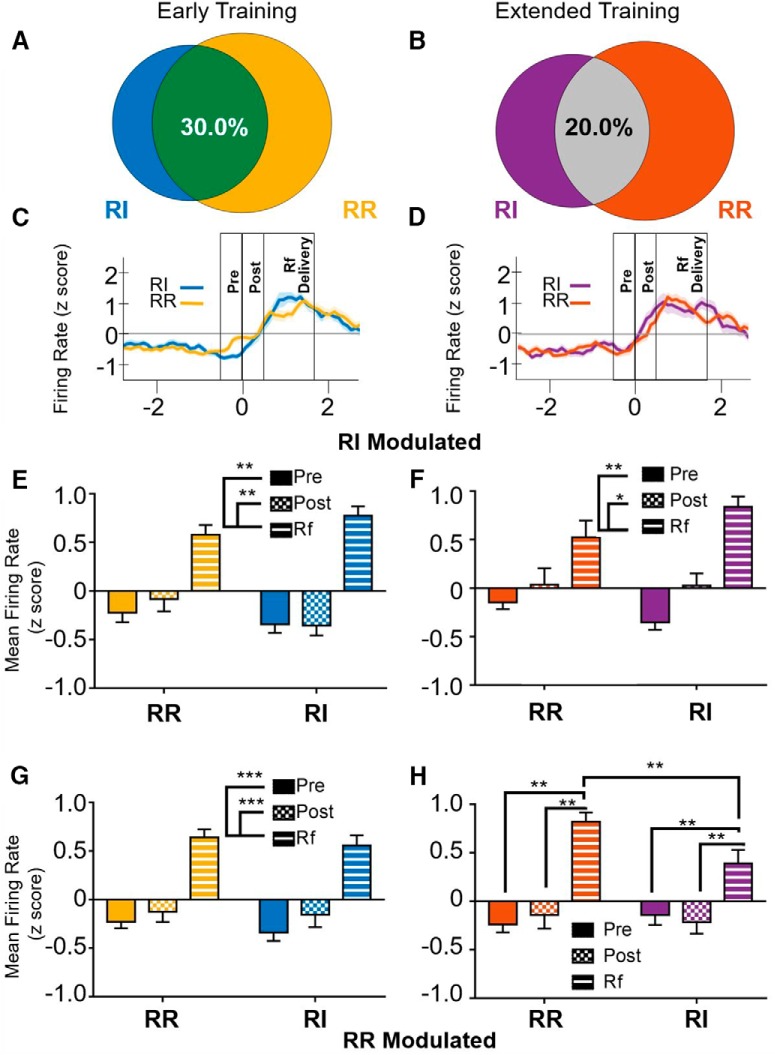
Activity patterns of cells with increased firing rates during reinforcer delivery. ***A***, At the early training time point, of cells that showed an increase in firing rate during either the RI or RR schedule, 30.0% exhibited elevated firing rates in both conditions. ***B***, At the extended training time point, 20.0% of cells that had increased firing rates during either the RI or RR schedule showed elevated firing rates in both conditions. ***C***, ***D***, Firing rate of cells showing an increase in firing surrounding a reinforced lever press epoch (i.e., goal directed on both the RR and RI levers) at the early training (***C***) or extended training (***D***) time points. Time 0 represents the time of lever press. Reinforcer delivery lasted for 1.7 s following lever press. ***E***, ***F***, Mean firing rates of cells showing an increase in firing rate during the RI schedule. ***E***, At the early training time point, RI-modulated cells showed similar activity patterns when responding on the RR schedule. ***F***, Similarly, at the late time point, RI-modulated cells showed similar activity patterns when responding on the RR schedule. ***G***, ***H***, Mean firing rates of cells showing an increase in firing rate during the RR schedule. ***G***, At the early training time point, RR-modulated cells showed similar activity patterns when responding on the RI schedule. ***H***, After extended training, cells that exhibited increased firing rates during reinforcer delivery on the RR schedule showed a similar pattern when responding on the RI schedule; however, they exhibited lower firing rates during the reinforcer delivery epoch on the RI (habit-promoting) schedule than on the RR schedule; **p < 0.01, ***p < 0.001.

**Figure 6.**
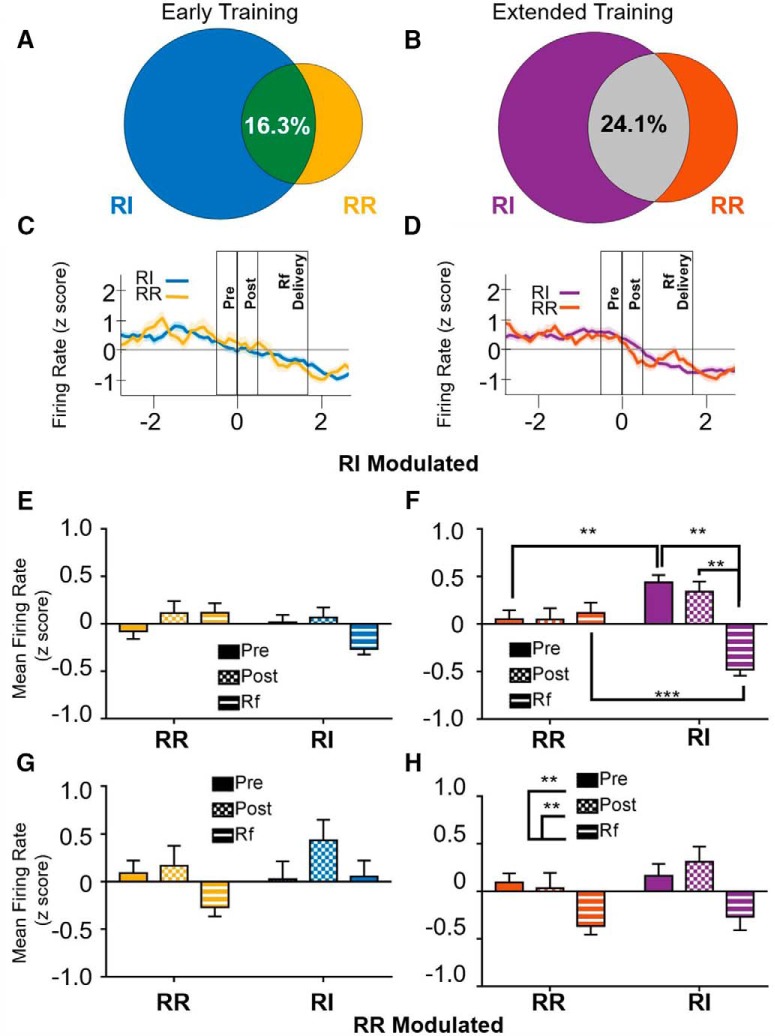
Activity patterns of cells with decreased firing rates during reinforcer delivery. ***A***, At the early training time point, 16.3% of cells that reduced firing rate during either the RI or RR schedule showed decreased firing rates in both conditions. ***B***, After extended training, 24.1% of cells that exhibited a decrease in firing rate during either the RI or RR schedule showed reductions in firing rates in both conditions. ***C***, ***D***, Firing rate of cells showing a reduction in firing rate surrounding a reinforced lever press epoch (i.e., goal directed on both the RR and RI levers) at the early training (***C***) or extended training (***D***) time points. Time 0 represents the time of lever press. Reinforcer delivery lasted for 1.7 s following lever press. ***E***, ***F***, Mean firing rates of cells showing reduced firing rates on the RI schedule. ***E***, At the early training time point, RI-modulated cells were not significantly modulated by epoch or by schedule. ***F***, In contrast, after extended training, RI-modulated cells showed a significant reduction in firing rate when responding on the RI schedule, exhibiting lower mean firing rates during the reinforcer delivery epoch than the pre- or post-press periods. However, cells modulated during the RI condition were not significantly modulated during responding on the RR condition. ***G***, ***H***, Mean firing rates of cells showing reduced firing rates on the RR schedule. ***G***, At the early training time point, RR-modulated cells were not significantly modulated by epoch or by schedule, similar to findings from RI-modulated cells. ***H***, Unlike cells reduced on the RI schedule, cells identified as modulated during responding on the RR schedule after extended training exhibited similar response patterns on both the RR and RI schedule; *p > 0.05, **p < 0.01, ***p < 0.001.

Through the use of the RR/RI model in which animals perform goal-directed and habitual responses within a single session, we examined how individual neurons were modulated during reinforcer delivery in both conditions. to determine whether the same neurons encode outcome during goal-directed actions and responses during habitual behavior, we assessed firing rates and the proportion of cells that were modulated during RR and RI. The proportion of cells modulated during outcome delivery was calculated for each time point, for both RI and RR. We then compared the activity of cells showing either significant increases (Fig. 5) or decreases (Fig. 6) during all epochs and schedules at the early or extended time points.

For IfL-C cells that exhibited increased firing rates during reinforcer delivery in either the RR or RI condition, 30.0% of those cells showed significantly increased firing rates in both conditions at the early training point (Fig. 5*A*), and only 20% were significantly increased in both conditions after extended training (Fig. 5*B*). A rmANOVA indicated that cells that were identified by d*'* as having significantly increased firing rates during the reinforcer delivery epoch when responding on the RI condition showed similar response patterns in the RR condition at the early training time point [no effect of schedule; *F*_(1,26)_ = 0.569, *p* = 0.457; Fig. 5*E*_j_]. A main effect of epoch was present [*F*_(2,52)_ = 53.974, *p* < 0.001], and *post hoc* comparisons indicate that firing rate was significantly higher during the reinforcer delivery epoch than either the pre- or post-press intervals (*p* < 0.01). While a significant interaction was observed, no differences between firing rates on the RR or RI schedules were observed. A similar finding was observed at the extended training time point (Fig. 5*F*). Firing rates of cells that were significantly modulated during the reinforcer delivery epoch on the RI schedule responded similarly in RR and RI conditions. No effect of reinforcement schedule was present at the early training time point [unequal distribution of variances; *F*_(1,21)_ = 0.532, p 0.474_k_] nor was there a significant epoch × schedule interaction [*F*_(1.957,41.107)_ = 2.83, *p* = 0.072]. A main effect of epoch was observed [*F*_(1.309,27.48)_ = 16.522, *p* < 0.01]. *Post hoc* analyses indicate that firing rate at the reinforcer delivery epoch was significantly higher than the pre- or post-press intervals (*p* < 0.01, *p* < 0.05, respectively).

For cells that exhibited increased firing rates during the reinforcer delivery epoch when responding on the RR schedule, findings at the early training time point were similar to the patterns observed for RI-modulated cells (Fig. 5*G*). No main effect of schedule was observed [*F*_(1,35)_ = 0.778, *p* = 0.384_l_] nor a significant schedule × time interaction [*F*_(2,70)_ = 0.103, *p* = 0.902]. A main effect of epoch was observed [*F*_(2,70)_ = 38.464, *p* < 0.001]. *Post hoc* analyses indicated that firing rates during the reinforcer delivery epoch are significantly higher than during the pre- or post-press intervals (*p* < 0.001). In contrast, at the extended training time point, cells that showed increased firing rates during the reinforcer delivery epoch on the RR schedule showed attenuated increases when responding on the RI schedule (Fig. 5*H*). A rmANOVA revealed no main effect of schedule [*F*_(1,29)_ = 2.916, *p* = 0.098_m_], but a significant main effect of epoch [*F*_(2,58)_ = 26.033, *p* < 0.001] and epoch × schedule interaction [*F*_(2,58)_ = 4.109, *p* < 0.05]. *Post hoc* analyses indicate that in both conditions, firing rates were higher during the reinforcer delivery epoch than during the pre- or post-press intervals (*p* < 0.01). However, firing rate of cells that were identified as modulated during the RR schedule showed lower firing rates when responding on the RI schedule than on the RR schedule (*p* < 0.01). Together, these findings suggest that cells that show increased firing rate during the reinforcer delivery epoch on the RI schedule are similarly modulated on the RR schedule at both the early and late training time points. This pattern was also observed at the early training time point for cells that exhibited significant increases in firing rate on the RR schedule, but this effect was attenuated at the extended training time point, suggesting that cells encoding outcome information on the RR (action-promoting) schedule are modulated to a lesser degree during the performance of habits.

For cells that exhibited a reduction in firing rate during the reinforcer delivery epoch in either the RR or RI condition, 16.3% of those cells showed significantly reduced firing rates in both conditions at the early training time point (Fig. 6*A*), and 24.1% were significantly reduced after extended training (Fig. 6*B*). For cells that were identified as significantly reduced during the reinforced delivery epoch on the RI schedule at the early training time point, rmANOVA indicated a main effect of schedule on firing rate [*F*_(1,34)_ = 5.394, *p* < 0.05; Fig. 6*E*_n_] such that firing rate was lower in the RI condition than the RR condition. No significant epoch [unequal variance in epoch measure; *F*_(1.59,54.056)_ = 0.741, *p* = 0.453] or schedule × epoch [*F*_(1.96,66.64)_ = 2.747, *p* = 0.072] effects were observed. In contrast, for cells with a reduced firing rate on the RI schedule after extended training, no effect of schedule was observed [*F*_(1,35)_ = 0.003, *p* = 0.955_o_], but firing rates were modulated by both task epoch [*F*_(2,70)_ = 6.532, *p* < 0.01] and by an epoch × schedule interaction [*F*_(2,70)_ = 14.355, *p* < 0.001; Fig. 6*F*]. *Post hoc* comparisons indicate that at the extended training time point, cells that exhibit reductions in firing rate on the RI schedule are not modulated on the RR schedule (no significant differences in firing rate based on task epoch; *p* > 0.05), while firing rates on the RI schedule were significantly lower during the reinforcer delivery epoch than either the pre- or post-press epochs (*p* < 0.01).

For cells with reduced firing rates during the reinforcer delivery epoch on the RR schedule, no main effects of schedule [*F*_(1,13)_ = 3.233, *p* = 0.095] or time [*F*_(2,26)_ = 2.862, *p* = 0.096_p_] were observed (Fig. 6*G*). No time × schedule interaction on firing rate was observed [*F*_(2,26)_ = 1.034, *p* = 0.370]. In contrast, at the extended training time point, task epoch-mediated firing rate [*F*_(2,42)_ = 7.431, *p* < 0.01; Fig. 6*H*_q_] such that firing rates were lower during the reinforcer delivery epoch than during the pre- or post-press intervals (*p* < 0.01). No main effects of schedule [*F*_(1,21)_ = 1.915, *p* = 0.18] or epoch × schedule interaction [*F*_(2,42)_ = 0.363, *p* = 0.698] were observed. Together, these findings suggest that at the extended training time point, cells that showed reductions in firing rates during reinforcer delivery on the RR schedule showed similar patterns of activity on the RI schedule. In contrast, after extended training cells that showed reductions in firing rate during the reinforcer delivery epoch on the habit-promoting (RI) schedule were not significantly modulated when responding on the RR schedule. Together with findings that fewer cells showed reductions in firing rate on the RR schedule than on the RI schedule (Fig. 4*B*, right panel, z = 2.258, *p* < 0.05), these findings suggest a potential mechanism by which the increase in firing rate observed during the reinforcer delivery epoch when mice are performing actions is lost during habitual behavior (Fig. 3*E*,*F*).

Because this task was self-paced, it was possible that magazine entries occurred during reinforcer delivery periods and may have contributed to results from the post-press and reinforcer delivery epochs. We did not exclude trials where animals entered the magazine during the reinforcer delivery period; however, our results indicate that for trials in which mice entered the magazine within 15 s of lever press, no significant difference in latency to enter the magazine following a reinforced lever press based on either reinforcement schedule [*F*_(1,271)_ = 0.330, *p* = 0.566_r_] or training time point [*F*_(1,271)_ = 1.058, *p* = 0.305], nor was a schedule × time point interaction present [*F*_(1,271)_ = 0.005, *p* = 0.944], suggesting that differences in latency to enter the magazine following a reinforced press do not drive observed differences in IfL-C activity. The overall mean latency to enter the magazine was 2.001 s (±0.203 s, SEM), which occurred outside of the reinforcer delivery epoch (Fig. 2*A*).

To determine whether schedule-mediated differences in firing rate were present when animals were not actively initiating a response or engaged in reward collection or consumption, we assessed firing rates from 1 s to 0.5 s before a lever press. Our results indicate that schedule did not significantly mediate firing rates at this baseline task epoch at either the early training (non-normal distribution; Wilcoxon test; z = 1.365, *p* = 0.172_s_) or the extended training time point (non-normal distribution; Wilcoxon test; z = 0.929, *p* = 0.353_t_). Further, no effect of schedule on firing rates were observed in the pre-press epoch (500 ms to press) at either time point (early: z = 0.185, *p* = 0.853_u_; extended: z = 0.015, *p* = 0.988_v_).

### Habitual reward seeking is associated with loss of modulation of IfL-C firing rate during isolated unreinforced presses

To determine whether increased firing rate following lever presses that were proceeded by outcome delivery during goal-directed actions was unique to reinforced presses (i.e., those presses that are followed by reinforcer delivery), we investigated whether unreinforced, isolated lever presses were associated with increased firing rate during habitual and goal-directed reward seeking. Here, only unreinforced which were separated from either another lever press or a magazine entry were included, representing 13.2% of all unreinforced presses. No differences in the proportion of trials included were observed based on either schedule [*F*_(1,51)_ = 1.557, *p* = 0.218_w_] or time point [*F*_(1,51)_ = 0.003, *p* = 0.955], nor was a time point × schedule interaction present [*F*_(1,51)_ = 0.102, *p* = 0.751].

**Figure 7.**
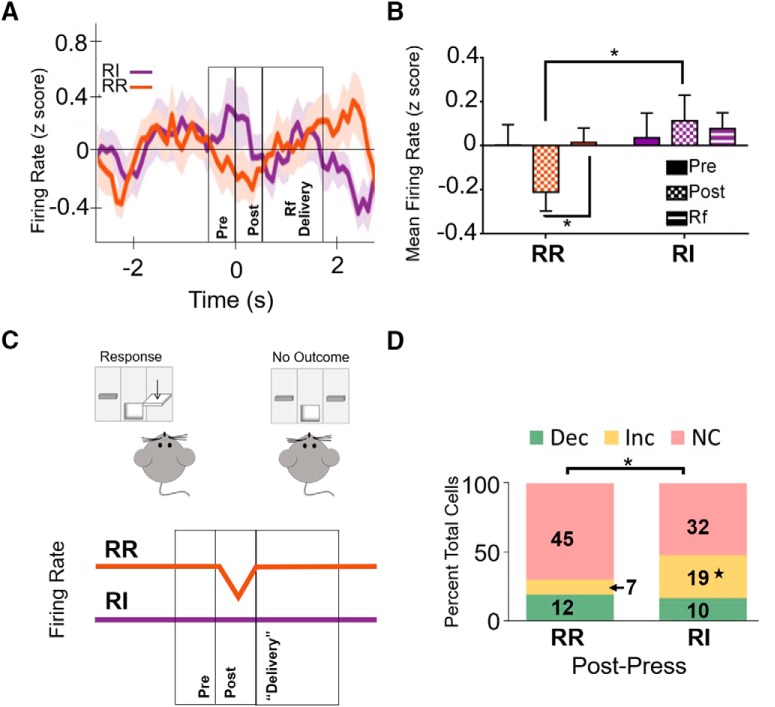
Firing rate following isolated, unreinforced lever presses. ***A***, Firing rate was differentially modulated based on reinforcement schedule following an isolated, unreinforced press at the extended training time point. Data represent mean ± SEM (indicated by shaded area for each line). Time 0 represents lever press. ***B***, After extended training, firing rate in the IfL-C during the post-press interval was significantly lower following unreinforced presses during the RR schedule than during the RI schedule. Firing post-press on the RR schedule was also significantly lower than firing during the epoch where reinforcer would be delivered during a reinforced press. ***C***, When mice are responding on an action-promoting (RR) schedule, unreinforced lever presses are followed by a reduction in IfL-C firing rate. In contrast, this modulation is attenuated when mice are responding on the habit-promoting RI schedule. ***D***, At the extended training time point, the distribution of significantly modulated cells was distinct between the RI and RR schedule during the post-press interval; *p < 0.05; Dec, decreased firing rate; Inc, increased firing rate; NC, no change in firing rate; ★ indicates significant differences in *z* score comparison of population proportions.

Analyses of isolated, unreinforced presses indicated an interaction between schedule and task epoch [*F*_(2,90)_ = 3.59, *p* < 0.05_x_], but no significant main effect of schedule [*F*_(1,45)_ = 2.152, *p* = 0.149] or interval [*F*_(2,90)_ = .615, *p* = 0.150]. Indeed, after extended training, firing rate during the post-press interval following unrewarded lever presses was higher on the RI (habit-promoting) schedule than on the RR schedule (Fig. 7*A*,*B*). There were also significant differences in the distribution of neurons during the post-press unrewarded epoch when mice were responding on the RI schedule compared to responding during the RR schedule (χ^2^ = 7.847, *p* < 0.05; Fig. 7*D*). Analysis further revealed that a greater proportion of cells exhibited a significant increase in firing rate during this task epoch in the RI condition compared to the RR condition (z = 2.7827, *p* < 0.01_y_), while no differences were observed in the proportion of unmodulated cells during this epoch (z = 0.3458; *p* = 0.726_z_). Unlike observations following extended training, there were no differences between the proportion of cells modulated during RR and RI early in training during the post-press epoch (χ^2^ = 0.217, *p* = 0.897; data not shown). These findings indicate response strategy mediates firing rate surrounding unreinforced lever presses, but in ways that are distinct from modulation of reinforced presses. In contrast to reinforced lever presses in which outcome delivery was associated with an increase in firing rate during goal-directed actions, unreinforced lever presses were followed by a reduction in firing rate in the epoch immediately following lever presses (Fig. 7*C*). This significant reduction in firing rate is absent during habits. Together, these findings indicated that neural activity following lever pressing is bidirectionally regulated during goal-directed actions to encode outcome information and that this modulation is selectively lost during habitual behavior.

### Lever press is associated with increased firing in IfL-C during outcome devaluation

**Figure 8.**
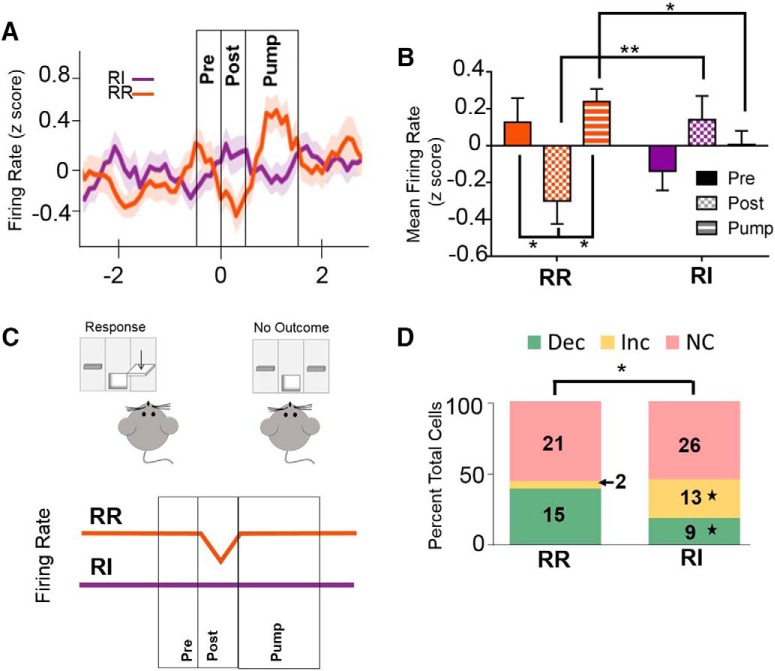
Habitual reward seeking was associated with loss of modulation in the post-press interval. ***A***, During an outcome devaluation test session, performed in extinction, IfL-C firing rate during the intervals surrounding a lever press was mediated by reinforcement schedule. Data represent mean ± SEM (indicated by shaded area for each line). Time 0 represents lever press. ***B***, During the post-press interval, neural activity was significantly lower during responding on the RR lever than the RI lever. IfL-C activity during the post-press epoch was significantly lower than the pre-press or the epoch where the reward delivery pump was on during responding on the RR schedule. ***C***, In an outcome devaluation test session, performed in extinction, IfL-C activity surrounding lever press is schedule dependent. When mice are responding on an action-promoting (RR) schedule, IfL-C activity is reduced immediately following lever press. In contrast, when mice are responding on a habit-promoting (RI) schedule, IfL-C activity is not modulated. ***D***, Significantly modulated cells were identified by d*'* analysis. At the extended training time point, the distribution of significantly modulated cells in the post-press interval was distinct between the RR and RI schedules. A greater number of cells showed a significant increase in firing rate in the post-press epoch during the RI schedule compared to the RR schedule, while a smaller proportion showed reductions in firing rate during this epoch; *p < 0.05, **p < 0.01; ★ indicates significant differences in *z* score comparison of population proportions.

To assess whether these altered firing rate patterns were present during tests of habitual reward seeking, firing rate was examined during outcome devaluation testing. To investigate firing rate during the pre-press, post-press and pump-on epochs, data were binned as described above (Fig. 8*B*). An assessment of the role of outcome devaluation on responding was performed during extinction, but the pump sound was delivered, and thus during the pump-on interval, the sound of the pump is presented, but no reinforcer is delivered. A rmANOVA on firing rate during these epochs indicated an epoch × schedule interaction [*F*_(2,168)_ = 6.357, *p* < 0.01_aa_]. No main effects of epoch or schedule were observed (*p* = 0.147 and *p* = 0.895, respectively). *Post hoc* comparisons indicated a significant reduction in firing during the post-press interval during goal-directed behavior versus the pre-press and pump-on intervals (*p* < 0.05). In addition, firing rate during the post-press interval was significantly lower during the RR test session than during the RI test session (*p* < 0.01). In contrast, firing rates during the reward delivery epoch were higher during the RR session than during the RI session (*p* < 0.05). These findings suggest that as in reinforced sessions, IfL-C activity is modulated by outcome information during goal-directed behavior, but that this modulation is absent during habits (Fig. 8*C*). Cells that showed significant modulation during the post-press and reward delivery epochs were identified by d′ analysis. A comparison of cells modulated during the post-press interval indicated differential distributions of cells modulated during the RR and RI schedules (χ^2^ = 9.058, *p* < 0.05; Fig. 8*D*). Comparison of cell populations indicated that a greater proportion of cells showed an increase in firing rate during the RI condition than during the RR condition during this task epoch (z = 2.6482, *p* < 0.01_ab_) while no differences in proportions of unmodulated cells were observed (z = 0.1014, *p* = 0.92). In addition to a greater proportion of cells showing increased firing rates, there was also a smaller proportion of cells showing a reduction in firing rate (z = 2.12, *p* < 0.05_ac_). In contrast, no differences were observed during the pump-on interval (χ^2^ = 0.1, *p* = 0.95; data not shown). Of note, no nondevalued test sessions were performed, and thus it is important to consider that nonspecific effects of satiety may contribute to the effects observed in the devaluation test session.

### IfL-C activity at lever press is required for the maintenance of habitual reward seeking

**Figure 9.**
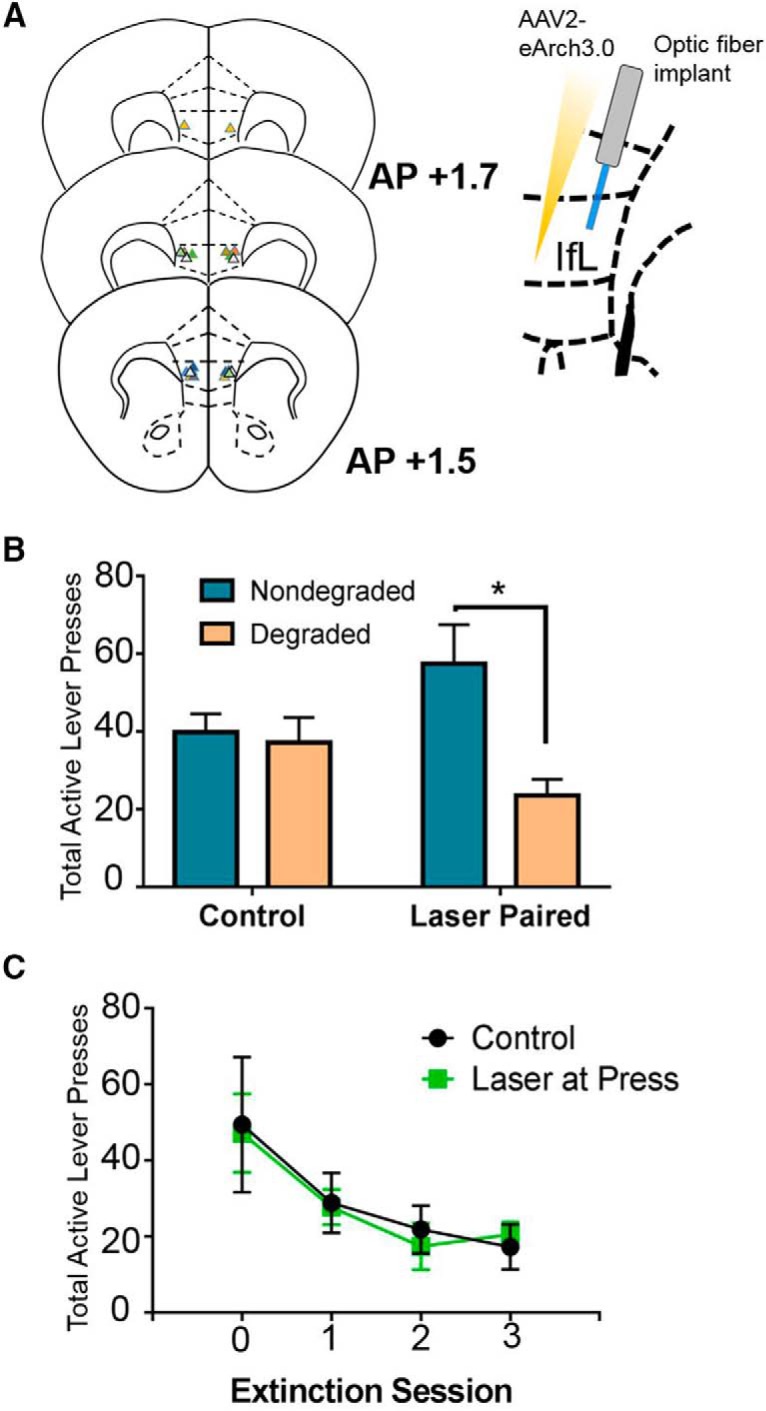
Optogenetic inhibition of IfL-C selectively impacted expression of habitual reward seeking. ***A***, Schematic showing placement of optic fibers and virus within the IfL-C. ***B***, Optogenetic inhibition of IfL-C selectively in the post-press epoch restored goal-directed behavior during a contingency degradation test. ***C***, Inhibition of IfL-C during the post-press epoch (at the same epoch shown to restore goal-directed actions) does not impact extinction learning; *p < 0.05. Data represent mean ± SEM.

To confirm that IfL-C firing at press during habitual behavior had a causal role in the maintenance of habitual behavior, mice were injected with a virus encoding the inhibitory opsin Arch-T and implanted with optic fibers targeting the IfL-C, and then trained to self-administer sucrose in the same RR/RI paradigm described above (Fig. 9*A*). Mice were assigned based on response rate during the RI intervals to one of three groups at test, “laser at press” where the laser was turned on for 0.5 s following a lever press, “laser unpaired” where the laser was turned on for 0.5 s that was explicitly unpaired with lever press or reinforcer delivery, or “no laser” where light was not delivered to inhibit the IfL-C. No differences were observed between the laser unpaired and no laser groups and thus these groups were collapsed into a single group (“control”). Results indicated an interaction between laser and test condition [rmANOVA contingency degradation × laser group interaction *F*_(1,8)_ = 10.825, *p* < 0.05; Fig. 9*B*_ad_]. A main effect of degradation [*F*_(1,8)_ = 14.824, *p* < 0.01] was present. No main effect of laser group was observed [*F*_(1,8)_ = 0.062, *p* = 0.809]. *Post hoc* analyses indicated that selectively inhibiting the IfL-C at press resulted in restoration of sensitivity to contingency degradation (i.e., a reduction in response rate during the degradation condition as compared to the nondegraded condition; *p* < 0.05), while mice in control conditions responded equally regardless of whether the contingency was degraded or intact (*p* = 0.643). Importantly, this effect does not appear to be a generalized reduction in responding as data are compared to a nondegraded test session in which mice were assigned to the same laser condition. In addition, examining how inhibition of IfL-C at lever press impacted the acquisition of extinction learning confirmed that reductions in responding during the contingency degradation test session were not mediated by facilitated extinction (Fig. 9*C*). While a main effect of session was observed [rmANOVA main effect of session *F*_(3,27)_ = 7.795, *p* < 0.001_ae_], there was no interaction with laser condition [*F*_(3,27)_ = 0.1104, *p* = 0.9533] or main effect of laser [*F*_(1,9)_ = 0.01719], indicating inhibition of IfL-C had no effect on extinction.

### Reward consumption results in alterations in firing rates in IfL-C

**Figure 10.**
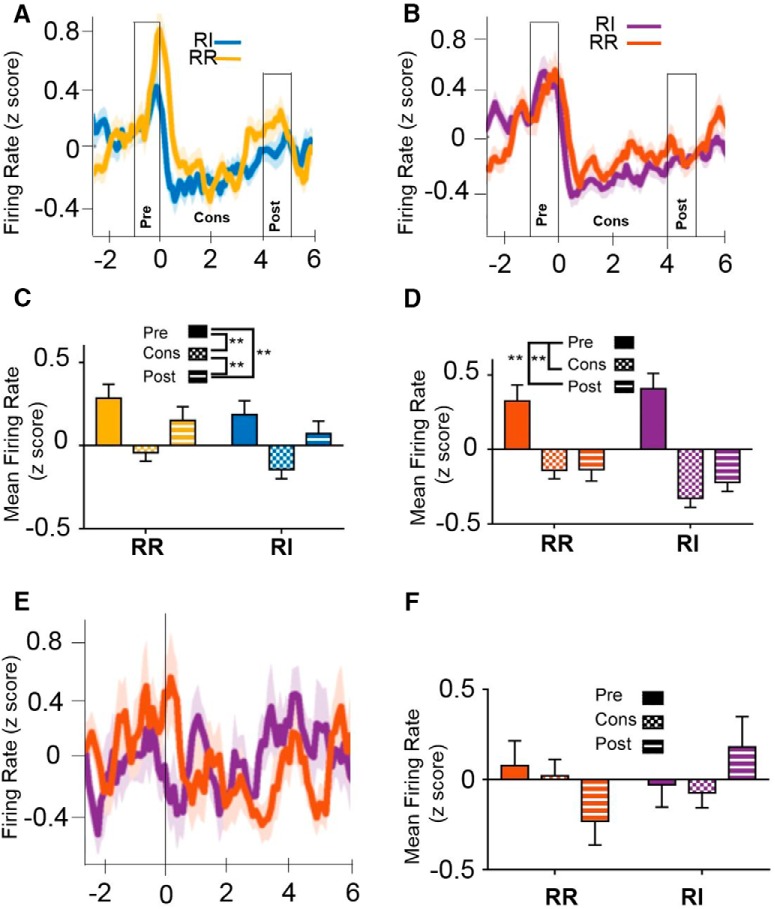
Consummatory behavior mediates firing rate in IfL-C independent of response strategy or reinforcement schedule. At the early training time point (***A***) and at the extended training time point (***B***), IfL-C activity is modulated across the onset of consummatory behavior. Time 0 represents time of entry (indicated by a beam break) into the magazine where the reinforcer was delivered and provides a putative indicator of initiation of consumption. The 0- to 4-s time period represents the putative consumption period. The one second interval before and after this period were considered the pre- and postconsumption intervals, respectively. ***C***, Binned means of firing rate at the early training period indicated that IfL-C firing rate during the consummatory period is significantly lower than mean firing rate pre- or postconsumption. ***D***, At the extended training time, firing rate in IfL-C during both the consumption and postconsumption interval was lower than the preconsumption interval. ***E***, During the outcome devaluation probe test, which was performed in extinction, IfL-C activity was not modulated during magazine entries, suggesting that magazine entry in the absence of consummatory behavior did not contribute to IfL-C activity. ***F***, No significant differences in IfL-C activity were observed during magazine entries during extinction. Data in represent the mean ± SEM indicated by shaded area for each line (***A***, ***B***, ***E***) or error bars (***C***, ***D***, ***F***); **p < 0.01.

In addition to independence from action-outcome contingencies, habits are less sensitive to perturbations in outcome value (Dickinson, 1985), suggesting that the encoding of rewards may be altered when behavior is habitual. To determine whether neural activity is altered during the reward, we examined neural firing during consummatory behavior (Fig. 10). To investigate firing rates across this period, data were binned into preconsumption (−1-0 s), consumption (0-4 s), and postconsumption (4-5 s). At the early training time point, a rmANOVA indicated a main effect of epoch [unequal distribution of variance; Greenhouse-Geissen correction; *F*_(1.577,137.224)_ = 8.324, *p* < 0.01_af_], but no effect of schedule [*F*_(1,87)_ = 3.026, *p* = 0.085] or schedule × epoch interaction [*F*_(1.794,156.1)_ = 0.435, *p* = 0.648; Fig. 10*A*,*C*]. *Post hoc* comparisons using Sidak’s correction indicate that firing during the consumption interval was significantly lower than either preconsumption (*p* < 0.01) or post consumption (*p* < 0.05), while firing rates did not differ between the pre- and postconsumption intervals (*p* = 0.731). Additionally, the consumption epoch was not significantly different from the postconsumption epoch (*p* = 0.366). At the extended training time point, rmANOVA again revealed a main effect of epoch [unequal distribution of variance; Greenhouse-Geissen correction; *F*_(1.424,121.063)_ = 22.05, *p* < 0.001_ag_], but no effect of schedule [*F*_(1,185)_ = 1.908, *p* = 0.171] or schedule × epoch interaction [*F*_(1.622,1137.8)_ = 2.274, *p* = 0.117; Fig. 10*B*,*D*]. *Post hoc* comparisons using Sidak’s correction indicate that firing rate during the consumption and postconsumption intervals were both significantly lower than the preconsumption interval (*p* < 0.01).

It is important to note that IfL-C activity modulation during magazine entry was restricted to reinforced sessions. During devaluation sessions, which were performed in extinction, when mice entered the magazine following pump sounds, IfL-C activity was not modulated (Fig. 10*E*,*F*). These data suggest that magazine entry alone is not sufficient to drive the suppression in IfL-C activity observed during putative consummatory epochs.

The pattern of neural activity during magazine entries that followed reinforced lever presses was also absent following unreinforced lever presses during the early and extended training sessions in which the RR and RI contingencies were intact. In contrast to the extinction test sessions, when mice entered the magazine following an unreinforced press at the early time point, firing rate was modulated by task epoch [*F*_(1.211,41.159)_ = 12.815, *p* < 0.001; data not shown_ah_] and a schedule × epoch interaction [*F*_(1.494,50.809)_ = 5.392, *p* = 0.013], but no main effect of schedule was observed [*F*_(1,34)_ = 0.473, *p* = 0.49]. Although no reinforcer was delivered during this period, task epochs were matched to those following reinforced press for comparison. *Post hoc* comparisons indicate that on the RI schedule, firing rates were higher during the matched “preconsumption” and “consumption” periods than during the “postconsumption” epoch (*p* < 0.01). In contrast, at the extended training time point, firing rates at magazine entry following an unreinforced press were modulated by both schedule [*F*_(1,30)_ = 4.839, *p* = 0.036_ai_] and task epoch [*F*_(1.064,31.929)_ = 4.362, *p* = 0.043. No significant interaction was observed [*F*_(1.208,36.236)_ = 1.498, *p* = 0.233]. *Post hoc* comparisons indicate that firing rate during the postconsumption was higher than firing rates during the preconsumption or consumption periods (*p* < 0.001). Of note, while latency to enter the magazine following a reinforced press did not differ based on reinforcement schedule or training time point, latency to enter the magazine following an unreinforced press was indeed modulated by a schedule × time point interaction [*F*_(1,390)_ = 5.023, *p* = 0.026]. *Post hoc* analyses indicate that latency to enter the magazine when responding on the RI schedule were significantly higher at the extended training time point (mean = 2.72 s, ± 0.338 SEM) than at the early training time point (mean ± SEM = 1.51 ± 0.144 s; *p* < 0.001). There was no significant difference between latency to enter the magazine after an unreinforced press at the early and late time point on the RR schedule, nor did these latencies significantly differ from latencies on the RI schedule.

In the next set of analyses, neurons that were significantly modulated during the consummatory period as compared to the pre- and postconsumption intervals were identified and split into groups based on the direction of modulation. At the early training time period, no differences in distribution of modulated cells were observed between the RR and RI conditions (χ^2^ = 2.391, *p* = 0.302). At this time point, in the RR condition, 30.6% exhibited increased firing rate, while 31.8% showed reduced firing rates during putative consumption. In the RI condition, 21.5% showed an increase in firing rate, while 40.9% showed a reduction in firing rate during the consummatory period. In contrast, at the extended training time point, a significant distribution in modulation was observed (χ^2^ = 7.605, *p* < 0.05). When mice were responding on an RR schedule, 30.2% exhibited increased firing rate, while 44.1% showed reduced firing rates during putative consumption. In the RI condition, 18.6% showed an increase in firing rate, while 65.1% showed a reduction in firing rate during the consummatory period.

**Figure 11.**
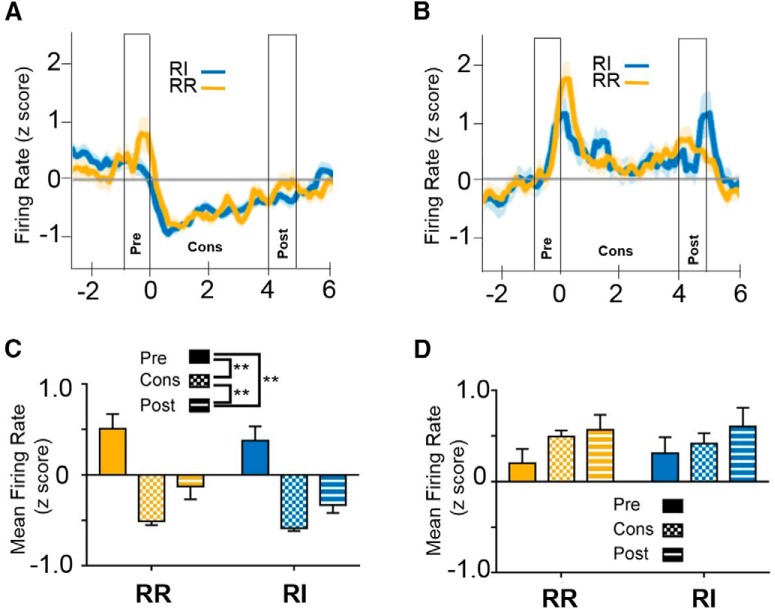
Firing rate during consummatory behavior at the early training time point. Cells that were significantly modulated during consumption were identified by d*'* analysis. ***A***, Firing rate of cells the exhibited a significant reduction in firing rate during consumption at the early training time point. ***B***, Firing rate of cells that increased their firing rate during consumption at the early training time point. Data presented in ***C***, ***D*** represent the mean ± SEM indicated by the shaded area for each line. ***C***, For cells that exhibited a reduction in firing rate, the rates were significantly lower during the consumption epoch than during the pre- or postconsumption epochs, and firing rates remained lower during the postconsumption period than during the preconsumption epoch. ***D***, In contrast, no significant differences were observed in the preconsumption, consumption, or postconsumption epochs in the cell that were identified as showing an increase in firing rate during the consummatory epoch. Data represent the mean ± SEM indicated by shaded area for each line (***A***, ***B***) or error bars (***C***, ***D***); **p < 0.01.

At the early training time point, a rmANOVA indicated that while there was a main effect of epoch on firing rate for cells showing a significant reduction during the consummatory period compared to baseline [unequal distribution of variance; Greenhouse-Geissen correction; *F*_(1.562,129.643)_ = 32.875, *p* < 0.001_aj_], there was not a schedule × epoch interaction [*F*_(1.562,129.643)_ = 0.374, *p* = 0.637; Fig. 11*A*,*C*]. A main effect of schedule was also present indicating higher firing rates overall in the RR schedule than RI, but this was not different across task epochs [*F*_(1,83)_ = 7.034, *p* < 0.01]. *Post hoc* comparisons indicated that the consumption epoch was significantly lower than the pre- and postconsumption epochs (*p* < 0.01) and that the pre- and postconsumption periods were significantly different (*p* < 0.01). In contrast, for neurons with firing rates during consumption that were higher than baseline, no significant differences were observed between the preconsumption, consumption, or postconsumption epochs [no main effect of epoch; *F*_(2,102)_ = 1.772, *p* = 0.175; Fig. 11*B*,*D*_a5_], nor was there any significant interaction with schedule [*F*_(2,102)_ = 0.222, *p* = 0.802] or main effect of schedule [*F*_(1,51)_ = 0.023, *p* = 0.881].

**Figure 12.**
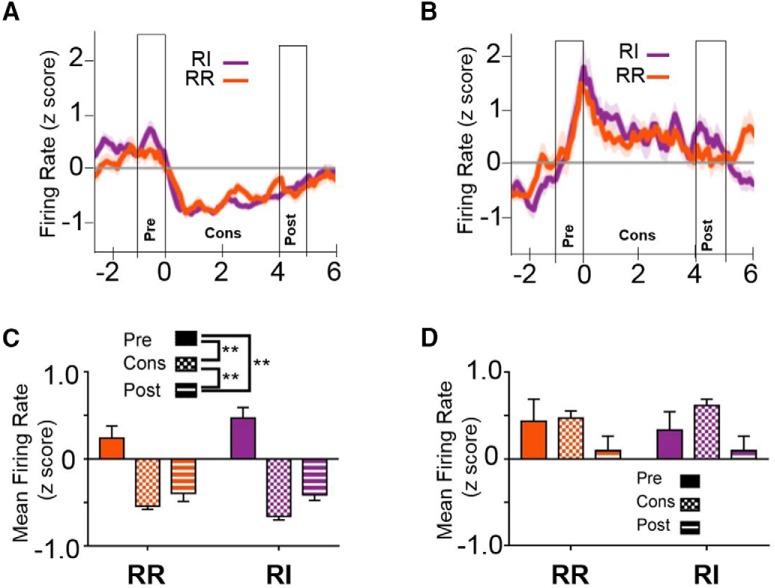
Firing rate during consummatory behavior at the extended training time point. ***A***, ***C***, Firing rate of cells that exhibited a reduced firing rate during the consummatory epoch at the late training period was not mediated by schedule, and there was no effect of schedule (i.e., RI and RR) across the preconsumption, consumption, or postconsumption intervals. ***B***, ***D***, For cells that exhibited an increase in firing rate during the consummatory epoch at the late training time point, there was no effect of schedule during any of the intervals. Data represent the mean ± SEM indicated by shaded area for each line (***A***, ***B***) or error bars (***C***, ***D***); **p < 0.01.

At the extended training time point, for cells showing a reduction in firing rate at the extended training time point, there was a main effect of task epoch [unequal variances; corrected with Greenhouse-Geisser; *F*_(1.395,153.4)_ = 54.616, *p* < 0.001_al_], but no schedule × epoch interaction [*F*_(1.395,153.4)_ = 1.506, *p* = 0.226] nor main effect of schedule [*F*_(1,110)_ = 0.678, *p* = 0.412; Fig. 12*B*,*D*]. *Post hoc* analyses indicated that the consumption period was significantly lower than both the pre- and postconsumption epochs (*p* < 0.001 and *p* < 0.01, respectively) and that the postconsumption period was significantly lower than the preconsumption period (*p* < 0.01). For cells showing significant increases in firing rate from baseline, no significant main effects of epoch [unequal variances; corrected with Greenhouse-Geisser; *F*_(1.461,68.665)_ = 1.305, *p* = 0.237_am_] or schedule [*F*_(1,47)_ = 1.345, *p* = 0.252], nor a schedule × time interaction were observed [*F*_(1.461,68.665)_ = 0.472, *p* = 0.566; Fig. 12*A*,*C*]. Together, these findings indicate that while firing rate in IfL-C is modulated by consummatory behavior, reward encoding is not mediated by response strategy or reinforcement schedule.

## Discussion

The results of the present study reveal that during performance of habitual behavior, information about action-outcome contingencies is differentially processed in the IfL-C. This is supported by the observation that during the performance of goal-directed actions, IfL-C appears to be bidirectionally regulated by outcome information. In particular, when responding on an action-promoting schedule, IfL-C activity is increased following reinforced lever presses during outcome delivery, and is reduced following an unreinforced press. In contrast, during the performance of habitual behavior, IfL-C is not modulated by responding or by outcome information. As habitual behavior is defined by independence from action-outcome contingencies or outcome value (Dickinson, 1985), it is of particular interest that our results reveal that during goal-directed behavior, the IfL-C encodes information about outcome availability independent of reward consumption and that this encoding appears to be absent or reduced during the use of habitual response strategies.

The loss of encoding of outcome information during habitual behavior may result from a rearrangement of IfL-C ensemble activity following extended training. At an early time point, modulated cells showed similar activity patterns when animals were responding on either an RR or RI schedule. After extended training, however, cells that were modulated during outcome delivery on an RR task showed attenuated modulation on the RI task. In addition, cells showing a reduction in firing rate on the RI schedule after extended training showed no modulation during any task epoch when animals were responding on the RR schedule. Together, these data indicate loss in overlap activity patterns across extended training and provide a potential mechanism by which the loss of outcome encoding during reinforcer delivery may occur.

It has previously been demonstrated that the absence of IfL-C activity restores goal-directed behavior and prevents the development and expression of habitual reward seeking (Coutureau and Killcross, 2003; Killcross and Coutureau, 2003; Smith et al., 2012). However, the reported role of the IfL-C in habitual reward seeking may appear to be at odds with the known role of the IfL-C in extinction. In particular, IfL-C appears to promote behavioral flexibility in extinction tasks and is necessary for acquisition and expression of extinction learning (Peters et al., 2009). This is in contrast to the ability of the IfL-C to promote behavioral inflexibility in habit tasks and support contingency-insensitive behaviors (Peters et al., 2009; Barker et al., 2014). The present findings suggest a mechanism by which IfL-C activity may mediate the expression of habitual behavior. During goal-directed actions, neural activity “ramps up” as information about outcome becomes available. In contrast, during habitual behavior, this increase in activity during outcome delivery is absent or attenuated. Because IfL-C activity is bidirectionally modulated by reinforcer delivery during goal-directed actions, this suggests the possibility that IfL-C activity is required following unreinforced lever presses to maintain responding during habitual behaviors when an action-outcome contingency is degraded. Alternatively, a reduction in IfL-C activity following an unreinforced press may be needed to accurately update action-outcome contingencies. While our findings demonstrate that inhibition of IfL-C immediately following lever press during a contingency degradation test prevented the expression of habitual behavior, it is not clear whether this is because IfL-C activity during this time point maintains habits, or because inhibition of IfL-C restores the ability to detect change in contingency.

In contrast to activity during outcome delivery, which our results demonstrate is response strategy-dependent, modulation in IfL-C activity during consumption was comparable across all conditions and included significant reductions in firing rate during consumption. Previous studies have reported modulation of prefrontal activity by consummatory behavior, and have suggested that PFC critically regulates consumption (Horst and Laubach, 2013). Interestingly, the observation in the present study that activity within IfL-C is reduced during consummatory behavior under both goal-directed and habitual conditions suggests that encoding of reward information in IfL-C is not altered during habitual reward seeking and that PFC control of consummatory behavior and/or processing of reward information is not impacted by response strategy.

These data provide novel insight into the neural computations performed by the IfL-C that integrate contingency and value information to drive inflexible, habitual reward seeking behavior. It is striking that modulation of IfL-C activity during consumption was comparable across all conditions, suggesting that processing of contingency and value information is dissociable in IfL-C as a function of response strategy. It should be noted that while loss of IfL-C function is sufficient to occlude habitual reward seeking (Coutureau and Killcross, 2003; Killcross and Coutureau, 2003; Smith et al., 2012), it is by no means the only structure necessary for habits (Yin et al., 2004; Lingawi and Balleine, 2012; Murray et al., 2015). Indeed, using a maze-based task, previous studies demonstrated that coordination between IfL-C and dorsolateral striatum activity is crucial for the development of inflexible behaviors (Smith and Graybiel, 2013). Given the role of IfL-C glutamatergic neurons in the regulation of response strategy (Smith et al., 2012), it is therefore likely that the IfL-C facilitates habitual reward seeking through its action at downstream targets. IfL-C targets multiple structures necessary for the regulation of response strategy selection, including subregions of the amygdala that are differentially involved in the acquisition and expression of habits (Lingawi and Balleine, 2012; Murray et al., 2015). Downstream targets of the IfL-C also include the nucleus accumbens in which IfL-C inputs are required for contingency-mediated behaviors such as appetitive extinction learning (Peters et al., 2008, 2009) and outcome selective Pavlovian-to-instrumental transfer (Keistler et al., 2015).

In summary, the results of the present study reveal that the IfL-C is critical for the expression of habitual behavior and suggest an online regulation of response strategy by IfL-C. We propose that the IfL-C tracks contingency information during goal-directed actions, and that this encoding is lost during habitual behavior. Gaining a greater understanding of the processes that cognitively bind outcomes to behavior have important implications for understanding neuropsychiatric illnesses in which the ability to flexibly regulate behavior is impaired. In addition, research using this habit model has the potential to provide important new insight into the neural networks that integrate contingency and value information to drive inflexible, habitual reward seeking behavior, and to identify novel treatment strategies for breaking maladaptive behavioral and cognitive habits.

## Synthesis

Reviewing Editor: Karen Szumlinski, University of California at Santa Barbara Decisions are customarily a result of the Reviewing Editor and the peer reviewers coming together and discussing their recommendations until a consensus is reached. When revisions are invited, a fact-based synthesis statement explaining their decision and outlining what is needed to prepare a revision will be listed below. The following reviewer(s) agreed to reveal their identity: Christina Gremel.

After consultation with 2 experts reviewers, we concur that the manuscript has great potential as it presents some very interesting findings but more revisions are required before the report will suitable for publication in eNeuro. We all agree that this additional revision requires no new experiments, but rather a revisit of the current data and some additional behavioral analyses to render the behavioral component more complete and related to the electrophysiological results. Specifically, Reviewer 1 suggested that the authors consider comparing the probability and latency to enter the port between the rewarded vs. unrewarded lever-presses to address the issue of whether or not the animals behave differently immediately following reward delivery vs. no reward delivery. This is the major revision that we agree would help solidify the paper and increase its impact. The reviewers have additional concerns (some relate to the insufficient description of behavior during the session, raised above) that are listed below:

Reviewer 1:

Basically, my concerns relate to the insufficient characterization of the behavior in the task, which ultimately limits the interpretation of the Ephys results.

- Arbitrary definition of epochs

First I have a question about the exact definition of the reinforcer delivery epoch. In the text, the authors define the reinforcer delivery period as the 1.7s of pump activation that immediately follows a lever press. However, in their figures the order of epochs appears to be: pre-press (-0.5 to 0s), post press (0 to 0.5s) and reinforcer delivery (0.5s to 1.7s). I'm not sure which one is correct. Does the reinforcer delivery epoch correspond to the last 1.2s of pump activation? Or the whole 1.7s (in which case it would encompass the post-press epoch). I assume it's the latter, if so the authors should clarify this in the figures and show the overlap between post-press and delivery epochs.

More generally, I still question the arbitrary choice of the reward delivery epoch. I appreciate that authors added information about latency from lever-press to port-entry, but that doesn't fully address the critical question: what happens behaviorally-speaking during these 1.7s, and why focus analyses of this one specific epoch? Is the assumption that this epoch is relevant because it is when the animals are hearing the pump? Another interpretation is that this period is relevant because animals are approaching the reward port. But if that's the relevant aspect then it would make more sense to time-lock this epoch to the animals entering the port. I believe this question (what's the rational for focusing analyses on this one particular epoch) is important because many critical results rely on this particular epoch.

- Analysis of non-reinforced lever presses

In their rebuttal and revised manuscript, the authors mention that isolated (non contaminated) lever presses represent 13% of all non-reinforced lever presses. That is surprisingly low and suggests that these non-reinforced lever press are not representative of the non-reinforced lever presses in general. So what are the animals doing when they press the lever and are not reinforced? Do they keep pressing in rapid succession until they hear the pump? Or do they check the reward port after a press, rewarded or not? What is the percentage of presses that have been excluded because they were immediately followed by a port entry? Assuming sufficient sample size, this last category of lever press could be useful to address the previous question (what drives Ephys responses during reward delivery -sound of pump or port approach). Is firing identical during reinforced and non-reinforced lever presses when both are followed by the same port entry behavior? Or conversely is firing different depending on whether or not the pump is activated, even if subsequent port approach is similar?

Moreover, assuming that subjects occasionally check the port after non-reinforced lever press, the comparison of reinforced vs nonreinforced action sequences would provide a better argument to conclude that “magazine entry in the absence of consummatory behavior does not contribute to IFL-C activity”. Currently, authors rely on the probe test after devaluation to reach that conclusion (Fig. 10 e-f) but this is not ideal since in that session the overall state is different (satiation). Comparing reinforced vs nonreinforced action sequences within a single session would also have the added benefit of tracking the same neurons in those 2 conditions.

I realize that this comparison might add to an already long manuscript but I believe a better characterization of the non-reinforced presses is necessary because the reported neural inhibition after non-reinforced presses is one of the main rational for the opto experiment. Moreover, as just mentioned, this comparison could replace the data presented in figure 10 e-f

- Incomplete outcome devaluation protocol - Missing test in valued condition

I appreciate that the authors explicitly acknowledge the absence of a probe test in valued condition. They now state that beyond outcome devaluation, general satiety can differentially impact RR vs RI responding. That is certainly true, but I my initial argument was more general. Whatever differences are observed between RR and RI responding (both behavioral and physiological) might be not due to satiety at all; RI and RR responses might simply have different sensitivity to extinction.

- Data presentation

I appreciate that authors now separately compare activity of excited or inhibited neurons across similar epochs in different schedules. However, when presenting those results (Fig 5 & 6) the authors present only the average activity during the different epochs and not the actual traces. Because the results rely on somewhat arbitrarily defined epochs (see above), it seems important to see the actual firing rate traces throughout the 6s period to really appreciate how the schedule affects responding (ex: compare the trace of RR modulated neurons in RR vs RI). These traces can be easily be added to the figures and do not require any more statistical analyses or changes in the text.

Likewise, for figure 7: the authors should consider presenting the firing rate traces throughout the 6s period (as they do for figure 8). Again, this is especially important because this neural inhibition following non-reinforced lever press in RR schedule (but not in RI) is the rational for the opto experiment.

Minor comments:

-Line 277: Section title “IfL-C encodes actions instead of outcomes during habitual behavior”.

The data support this claim.

-Line 420: Section title “Habitual reward seeking is associated with modulation of IfL-C firing rate during isolated unreinforced presses”.

Isn't it the opposite: goal-directed reward seeking is modulated during unreinforced presses?

(See a few lines below: “Together, these findings indicated that neural activity following lever pressing is

bidirectionally regulated during goal-directed actions to encode outcome information, and that this

modulation is selectively lost during habitual behavior)

Reviewer 2:

The authors have largely addressed my concerns and added discussion limiting the interpretation of their findings where appropriate. They may want to consider adding the distributions of latencies they now report; as it may strengthen their findings- or limit analyses to subjects that actually show a head entry following reinforcer delivery.
